# A new technique for predicting intrinsically disordered regions based on average distance map constructed with inter-residue average distance statistics

**DOI:** 10.1186/s12900-019-0101-3

**Published:** 2019-02-06

**Authors:** Takumi Shimomura, Kohki Nishijima, Takeshi Kikuchi

**Affiliations:** Department of Bioinformatics, College of Life Sciences, Ritsumeikan University, 1-1-1 Nojihigashi, Kusatsu, Shiga 525-8577 Japan

**Keywords:** Intrinsically disordered protein, Average distance map, PrDOS, DISOPRED, Biomine

## Abstract

**Background:**

It had long been thought that a protein exhibits its specific function through its own specific 3D-structure under physiological conditions. However, subsequent research has shown that there are many proteins without specific 3D-structures under physiological conditions, so-called intrinsically disordered proteins (IDPs). This study presents a new technique for predicting intrinsically disordered regions in a protein, based on our average distance map (ADM) technique. The ADM technique was developed to predict compact regions or structural domains in a protein. In a protein containing partially disordered regions, a domain region is likely to be ordered, thus it is unlikely that a disordered region would be part of any domain. Therefore, the ADM technique is expected to also predict a disordered region between domains.

**Results:**

The results of our new technique are comparable to the top three performing techniques in the community-wide CASP10 experiment. We further discuss the case of p53, a tumor-suppressor protein, which is the most significant protein among cell cycle regulatory proteins. This protein exhibits a disordered character as a monomer but an ordered character when two p53s form a dimer.

**Conclusion:**

Our technique can predict the location of an intrinsically disordered region in a protein with an accuracy comparable to the best techniques proposed so far. Furthermore, it can also predict a core region of IDPs forming definite 3D structures through interactions, such as dimerization. The technique in our study may also serve as a means of predicting a disordered region which would become an ordered structure when binding to another protein.

**Electronic supplementary material:**

The online version of this article (10.1186/s12900-019-0101-3) contains supplementary material, which is available to authorized users.

## Background

Anfinsen’s discovery [1] that a protein exhibits its specific function through its own specific 3D-structure under physiological conditions dominated protein research for many years. However, proteins without specific 3D-structure under physiological conditions, known as intrinsically disordered proteins (IDPs), were later recognized [2, 3].

Dunker et al. were the first to apply bioinformatics techniques to the study of IDPs [4] by developing a program to predict regions with no defined 3D structure in a protein sequence, called PONDR [5]. Afterwards, other prediction programs were developed [3].

In such studies, a database of information on IDPs is indispensable. Among the various databases, DisProt [6] and IDEAL [7, 8] are widely used for IDP studies.

Surprisingly, IDPs exhibit their functions through interactions with another protein despite their lack of well-defined structures. A partial region with no well-defined 3D-structure is called an intrinsically disordered region (IDR). An example is cyclic-AMP response element-binding protein (CREB). This protein contains a partially disordered region which becomes ordered upon interaction with its co-activator, CREB-binding protein (CBP), thereby permiting its function.

Observations suggest that hydrophilic residues in IDRs are abundant and hydrophobic residues are scarce [4]. In addition, many simple repetitive sequences are found in IDRs [9]. Another remarkable property is that IDPs are abundant in eukaryotes [10]. A non-DNA binding domain sequence in a transcription factor sometimes contains disordered or unknown regions with many IDRs. Of the human transcription factors, 60% show this property [11]. IDPs with long disordered regions (> 30 residues) are found in about one third of eukaryotic proteins [10].

There is obvious difficulty in trying to determine their 3D-structures with techniques such as X-ray crystallography and NMR analysis. Therefore, research has focused on developing various techniques to predict IDRs in an amino acid sequence based on the sequence alone. These methods rely on various measures like amino acid propensity, secondary structure propensity or amino acid contact potential with various techniques such as support vector machine, neural net and so on [3]. In the CASP10 experiment, 28 groups submitted models to predict IDRs. While the models made accurate predictions to some degree, the improvement in accuracy was still considered slow [12].

Over the years, we have employed methods based on inter-residue average distance statistics to predict the folding mechanisms of various ordered proteins. We developed these techniques further to predict domain regions in a protein sequence. Our results indicate that the folding initiation sites of ordered proteins can be successfully predicted with a technique based on inter-residue average distance statistics and the information of conserved hydrophobic residues [13–17].

A method called DICHOT was developed [18] to predict the compact regions of a protein. This method assumes that a structural domain is ordered and thus an intrinsically disordered region (IDR) must be outside these compact regions.

Since our technique can predict a compact region in a protein sequence, the possibility of applying our technique to IDPs is interesting. The present study proposes a new technique for predicting IDRs from their sequence based on a map derived from the inter-residue average distance statistics, an average distance map (ADM).

## Methods

### Data set

The proteins examined in this study included completely ordered proteins, partially ordered proteins, and completely disordered proteins. In total, 160 completely ordered proteins with 60 to 219 residues were selected from the PDB, as shown in Table 1. In addition, 129 partially disordered and 74 completely disordered proteins were selected from DisProt [6]. The proteins were assigned to one of eight groups according to the number of residues in the proteins, as shown in Table 1. These proteins are summarized in Additional file 1. From each of the 8 groups, 20 proteins were selected for further study. We picked proteins from as wide a variety of sources as possible. In this study, we do not examine extremely large and complicated proteins: Proteins with a maximum of two domains are used as targets.

**Table 1:** The 160 completely ordered proteins collected from the PDB are shown

	Range of number of residues in a protein							
	60-79	80-99	100-119	120-139	140-159	160-179	180-199	200-219
Number of 100% ordered proteins	20	20	20	20	20	20	20	20
Number of 100% disordered proteins	8	12	20	7	5	10	4	8
Number of partially disordered proteins	10	14	16	9	20	21	19	20

The 129 partially disordered and 74 completely disordered proteins collected from DisProt are also shown [6]. Each protein is assigned to one of 8 groups based on the protein’s number of residues, with numbers ranging from 60 to 219 residues

### Average distance map (ADM) analysis

An average distance map (ADM) is constructed in a similar way as a contact map. For an ADM, as with a contact map, a plot is made on a map for a protein when the average distance of a pair of residues is less than a certain threshold. That is, an ADM is constructed from only the amino acid sequence of a given protein. A region forming a cluster of plots (pairs of residues) on a map predicts a portion with short distances between the residues in the native structure of a protein. Such regions correspond well to structural domains in proteins [19, 20] and also correspond to structurally compact regions in the early stage of protein folding. The combination of ADMs and information regarding evolutionally conserved hydrophobic residues has been amply demonstrated to predict the folding mechanisms of various proteins [13–17].

#### Calculations of the inter-residue average distances in proteins

The inter-residue average distance was calculated as the distance between the Cα atoms of residues in a protein whose 3D structure is known. We define a range as the distance between two residues along the sequence of a given protein. The range *M* = 1 is defined as 1 ≦ *k* ≦ 8, where *k* = |*i* − *j*| for *i*-th and *j*-th residues along the amino acid sequence. In the same way, the respective ranges *M* = 2, 3, 4 were defined as 9 ≦ *k* ≦ 20, 21 ≦ *k* ≦ 30, 31 ≦ *k* ≦40, and so on. The average value of the inter-Cα-atom distances for a pair of residue types in every range was calculated [19].

#### Construction of an ADM

As previously mentioned, the ADM for a protein is constructed using only the sequence information. A plot is made on a map when the average distance of a pair of residues within the range M is less than a certain threshold, and a threshold is defined for every range. The set of threshold values is determined in such a way as to reproduce the whole plot density of the contact map constructed from the 3D structure of a protein (real distance map, RDM) [19]. The values for the whole plot density of RDMs follow the formula, ${\uprho}_{av}=\frac{C}{N}$, where ρ_*av*_ is the plot density, *N* is the total number of residues for a given protein, and C is an adjustable constant [19]. It has been shown that C = 36.12 approximately reproduces the whole plot density of the RDM for a protein with an 15-Å cutoff [19]. We use this value in the present study.

The threshold value of the average distances in the range M to construct the ADM for a given protein is determined so as to reproduce the value of ${\uprho}_{av}=\frac{C}{N}.$ The number of pairs of residues in a range M to be plotted on an ADM obeys the following equation:

$$ \mathrm{P}{\left(\mathrm{M}\right)}_C=\left(\frac{D}{M}\right)P(M), $$

where P(M)_C_ is the number of residue pairs to be plotted, which should be the number of residue pairs with an average distance less than the threshold in the range M. P(M)t is the number of all residue pairs with statistically significant values for the average distances in the range M [19]. D is a parameter to adjust the plot density of the ADM closer to the value of ${\uprho}_{av}=\frac{C}{N}.$

#### Analysis of the ADM

A constructed ADM is analyzed by the following procedure.

##### 1. Calculation of the plot density differences

**Fig. 1:**
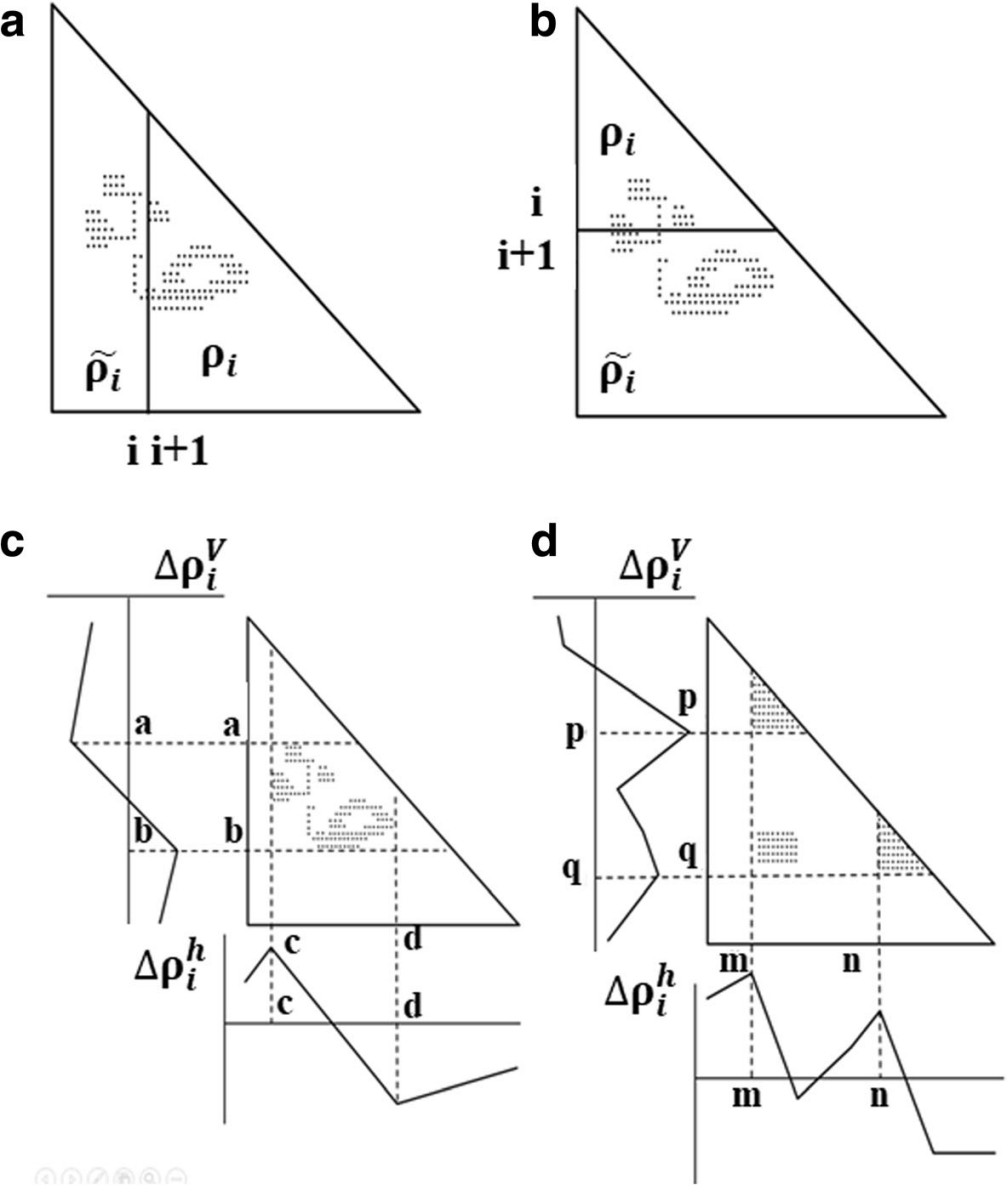
Schematic drawing of a map divided by a line parallel to the y-axis at the *i*-th residue (**a**) and divided by a line parallel to the x-axis at the *i*-th residue (**b**). The density of plots in the trapezoidal part and the triangular parts are denoted by *ρ*_*i*_ and ${\overset{\sim }{\rho}}_i$, respectively. **c** Schematic drawing of a map with some plots. A peak and a valley appear at the boundaries of a highly dense region of plots. This map predicts that many plots will form between the segments *a*–*b* and *c*–*d*. **d** Hypothetical map with two compact areas near the diagonal along with the horizontal and vertical scanning plots. This map predicts the existence of two domains at the regions *p*–*q* and *m*–*n*. We define η as a measure of the compactness of the region, namely, $\Delta {\uprho}_p^h+\Delta {\rho}_q^v$ or $\upeta =\Delta {\uprho}_m^h+\Delta {\rho}_n^v$

Suppose that an ADM is divided into two parts by a line parallel to the y-axis at the i-th residue or by a line parallel to the x-axis at the i-th residue as shown in Fig. 1 (a) and (b). Then, let us define *ρ*_*i*_ and ${\overset{\sim }{\rho}}_i$as the plot density of the triangular and trapezoidal parts, respectively. The plot density difference is defined as $\Delta {\uprho}_i={\rho}_i-{\overset{\sim }{\rho}}_i$.

The values of the plot density difference, Δρ_*i*_, are calculated from residues 1 to the total number of residues in a given protein. The plots obtained by the line parallel to the x-axis is called vertical scanning and those obtained by the line parallel to the y-axis is called horizontal scanning. *v* of $\Delta {\uprho}_i^v$and *h* of $\Delta {\uprho}_i^h$denote the vertical and the horizontal divisions of a map, respectively. In Fig. 1(c), the schematic drawing of the vertical and horizontal scanning plots of ADM is presented.

##### 2. Detecting the boundaries of a compact region

The existence of a peak and a valley in a scanning plot reflect a sudden change in the plot density values on a map. Figure 1(c) depicts a schematic example of the horizontal scanning plot of $\Delta {\uprho}_i^h$from 1 to *N*, and at the bottom of the figure, a peak and a valley appear at *c* and *d*, respectively, indicating a large change in plot density values. In the same way, a peak and a valley appear at *a* and *b*, respectively (shown the left of the figure), in the vertical scanning plot of $\Delta {\uprho}_i^v$. The boundary of a compact region on a map can be detected as a highly dense region of plots with a peak and a valley appearing in the horizontal and vertical scanning plots of density differences.

##### 3. Predicting the location of a compact region

The positions of peaks in scanning plots can define a possible compact region on an ADM. Figure 1(d) illustrates a hypothetical ADM with two compact regions near the diagonal. The horizontal and vertical scanning plots show the peaks at residues m and n and residues p and q, and these regions m-p and n-q on the map can be predicted as possible compact regions in a given protein. Furthermore, we use $\upeta =\Delta {\uprho}_m^h+\Delta {\rho}_n^v$ as a measure of the compactness of m-p [19].

## Results

### Properties of predicted ADM plots for completely ordered, completely disordered, and partially disordered proteins

**Fig. 2:**
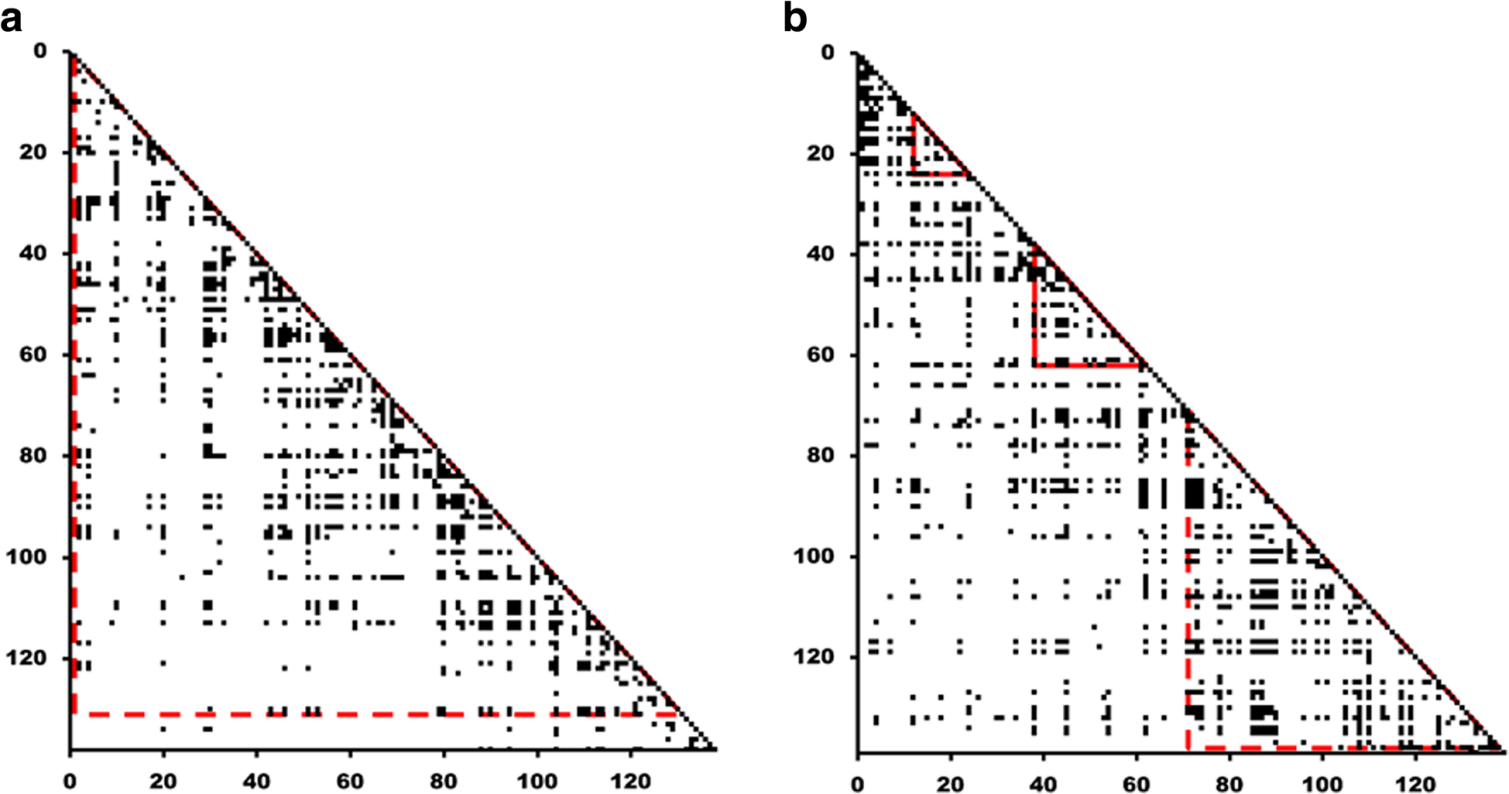
**a** ADM for an example of a completely ordered protein, arsenate reductase from *E. coli* (PDBID:1S3D). **b** ADM for an example of completely disordered protein, protein umuD from *E. coli* (DisProt code: DP00626). A region enclosed by a red triangle denotes a region with high η value, that is, a predicted compact region

Examples of ADMs for a completely ordered protein, arsenate reductase from *E. coli* (PDB ID: 1S3D), and a completely disordered protein, protein umuD from *E. coli* (DisProt code: DP00626), from the DisProt database, are shown in Fig. 2(a) and 2(b). As seen in Fig. 2, discriminating between ordered and disordered proteins by just glancing at the ADMs is difficult. Therefore, we analyze the ADM-plot density of the data set proteins in Table 1 in detail.

**Fig. 3:**
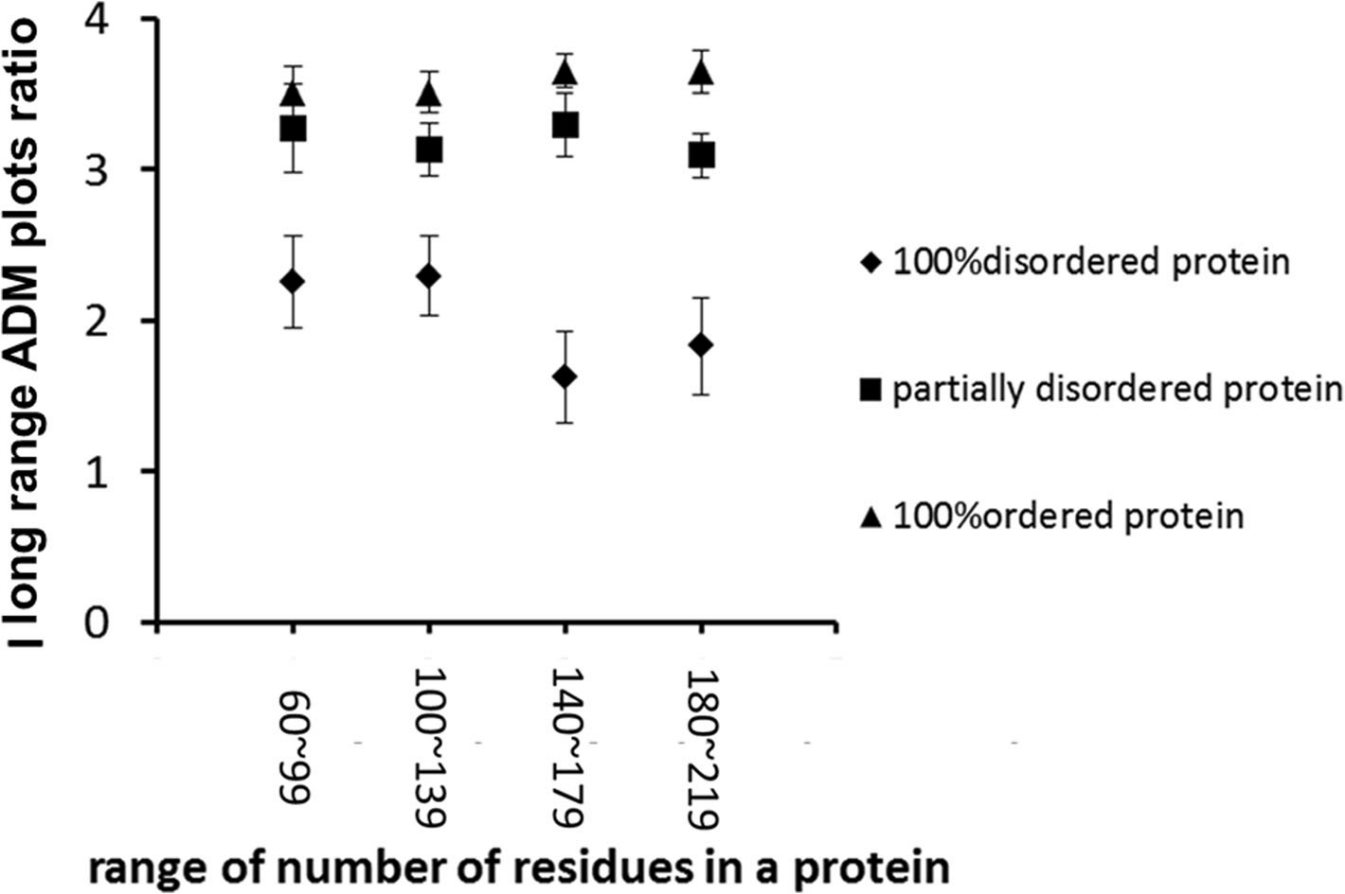
Plot of the average long-range ADM plot ratio values versus the window widths of 40 residues in proteins. A triangle, a rectangle, and a diamond denote completely ordered, partially disordered, and completely disordered proteins, respectively. “ADM plots ratio” refers to the ratio of the number of plots to the total number of residues in a given protein. Ted by more than 8 residues are counted in the ADM plot ratio calculation. An error bar is presented at each point, denoting the standard error value

Figure 3 shows the average of long-range ADM-plot density values for various sizes of completely ordered, completely disordered, and partially disordered proteins. ADM-plot density means the ratio of the number of plots on the ADM for a given protein to the total number of residues in this protein. We call this value “ADM-plot ratio”. The term “long-range” means that residue pairs that are separated by more than 8 residues are counted in the ADM-plot ratio calculation. In Fig. 3, one can see that the average values for completely ordered proteins tend to be the highest, those for partially disordered proteins intermediate, and those for completely disordered proteins the lowest. The ADM-plot ratio for long-range pairs can therefore be an indicator for IDRs (intrinsically disordered regions).

**Fig. 4:**
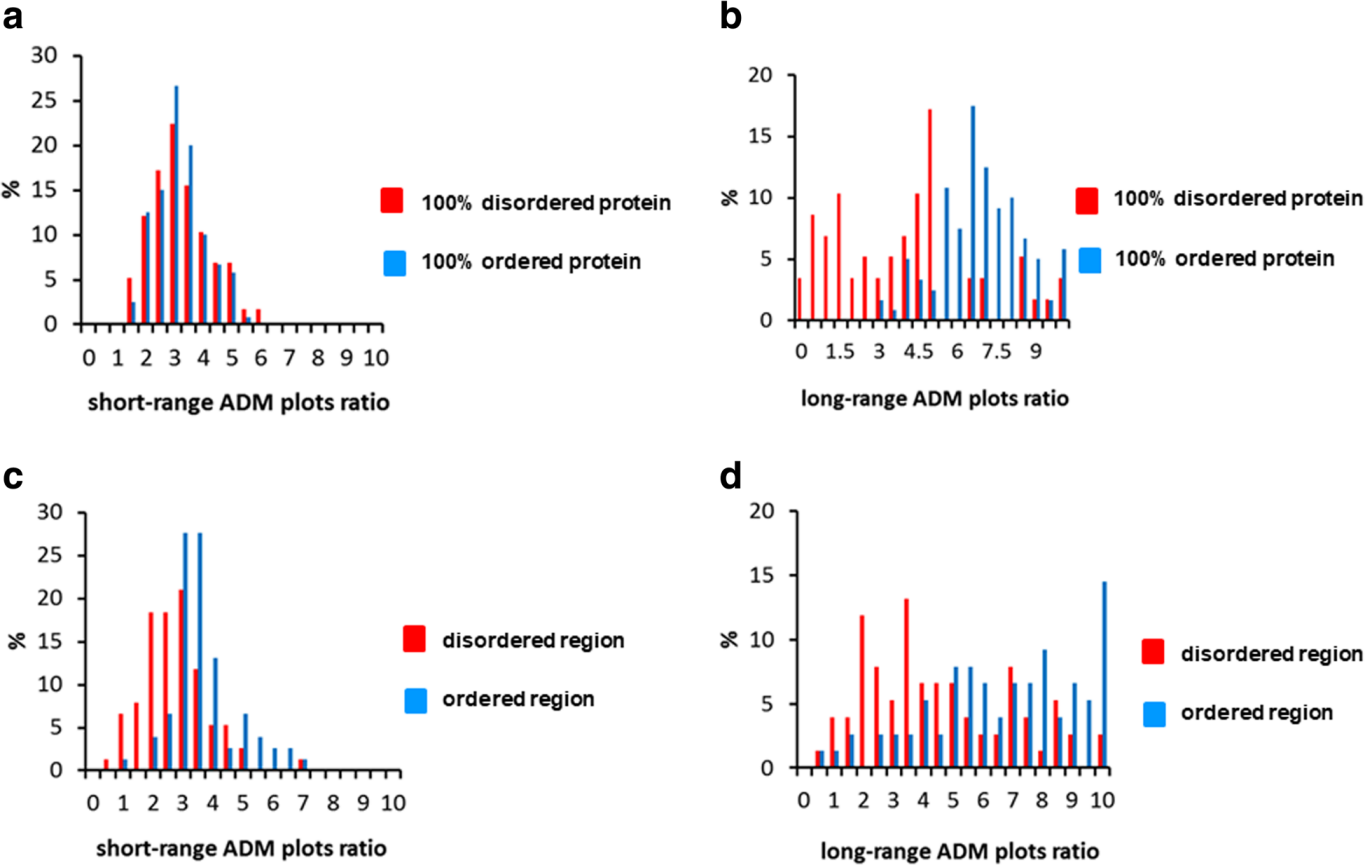
**a** Histogram of the number of completely disordered and completely ordered proteins versus short-range ADM plot ratio. “Short-range” refers to a plot on an ADM formed by a pair of residues separated by fewer than 9 residues along a given sequence. This histogram indicates the ratio (%) of the number of completely disordered or completely ordered proteins to the total number of proteins. **b** Histogram of the number of completely disordered and completely ordered proteins versus the long-range ADM plot ratio. “Long-range” refers to a plot on an ADM formed by a pair of residues separated by more than 8 residues along a given sequence. This histogram indicates the ratio (%) of the number of completely disordered or completely ordered proteins to the total number of proteins. **c** Histogram of the number of disordered and ordered regions of partially disordered proteins versus the short-range ADM plot ratio. **d** Histogram of the number of disordered and ordered regions of partially disordered proteins versus the long-range ADM plot ratio

Next, more detailed characteristics of the ADM-plot ratio are examined. Figure 4(a) presents a histogram of the number of completely disordered and completely ordered proteins versus the short-range ADM-plot ratio. Here, “short-range” refers to a plot on the ADM formed by a pair of residues separated by fewer than 9 residues along a given sequence. This histogram indicates the ratio (%) of the number of proteins to the total number of proteins. The average value of the short-range ADM-plot ratio for all the completely disordered proteins is 3.50, and the value for all the completely ordered proteins is 3.46. That is, there is no big difference in the tendencies between completely ordered and completely disordered proteins.

The same histogram for a long-range ADM-plot ratio is presented in Fig. 4(b). Again, “long-range plot” refers to a plot formed by a pair of residues separated by more than 8 residues along a given sequence. The average value of the long-range ADM plot ratios for all the completely disordered proteins is 4.18 and for all the completely ordered proteins is 7.15. Thus, there is a difference in tendencies between completely ordered and completely disordered proteins, namely, an ordered protein tends to show a high long-range ADM-plot ratio. We suggest that the long-range ADM-plot ratio discriminates between a disordered region and an ordered region. Figures 4(c) and (d) show figures analogous to Figures 4(a) and (b) for ordered and disordered regions in partially disordered proteins. For a partially disordered protein, the same calculation of the ADM-plot ratio is performed for both ordered and disordered region, and the values of the ADM-plot ratio are presented in Figs. 4(a) and (b).

Figures 4(c) and (d) suggest a similar tendency, with the long-range ADM-plot ratio presented in Fig. 4(b). From Fig. 4(b), the average value of the long-range ADM-plot ratio for all the disordered regions is 4.75 and for all the ordered regions is 7.33. In comparison, the average value of the short-range ADM-plot ratio for all the disordered regions is 3.00 and for all the ordered regions is 3.90 [Fig. 4(c)]. It is interesting that Fig. 4(c) indicates a higher short-range ADM-plot ratio in the ordered region. This tendency does not appear for the short-range ADM-plot ratio in completely ordered and completely disordered proteins. This result might suggest that discriminating between a disordered region and an ordered region becomes clearer when a partial segment is the focus. These results suggest that the tendency of the long-range ADM-plot ratio between completely ordered and completely disordered proteins is same as that for partially disordered proteins. However, the tendency of the short-range ADM-plot ratio observed in a completely disordered protein and a completely ordered protein is different from that in partially disordered proteins. This suggests that the tendency of the shorter-range ADM-plot ratio in long-range plots for completely disordered proteins and completely ordered proteins is different from that for partially disordered proteins. Therefore, in order to identify a disordered region in a partially disordered protein sequence, the effect of the relatively shorter-range ADM-plot ratio in long-range plots should be incorporated efficiently.

To extract only relatively short-range effects, we took ADM plots of pairs of residues separated by fewer than 30 residues along the sequence of the protein under consideration.

### The procedure for identifying a partially disordered region

Next, we aimed to develop a technique for predicting disordered regions in a protein sequence.

#### Determining the disorder probability of a residue

**Fig. 5:**
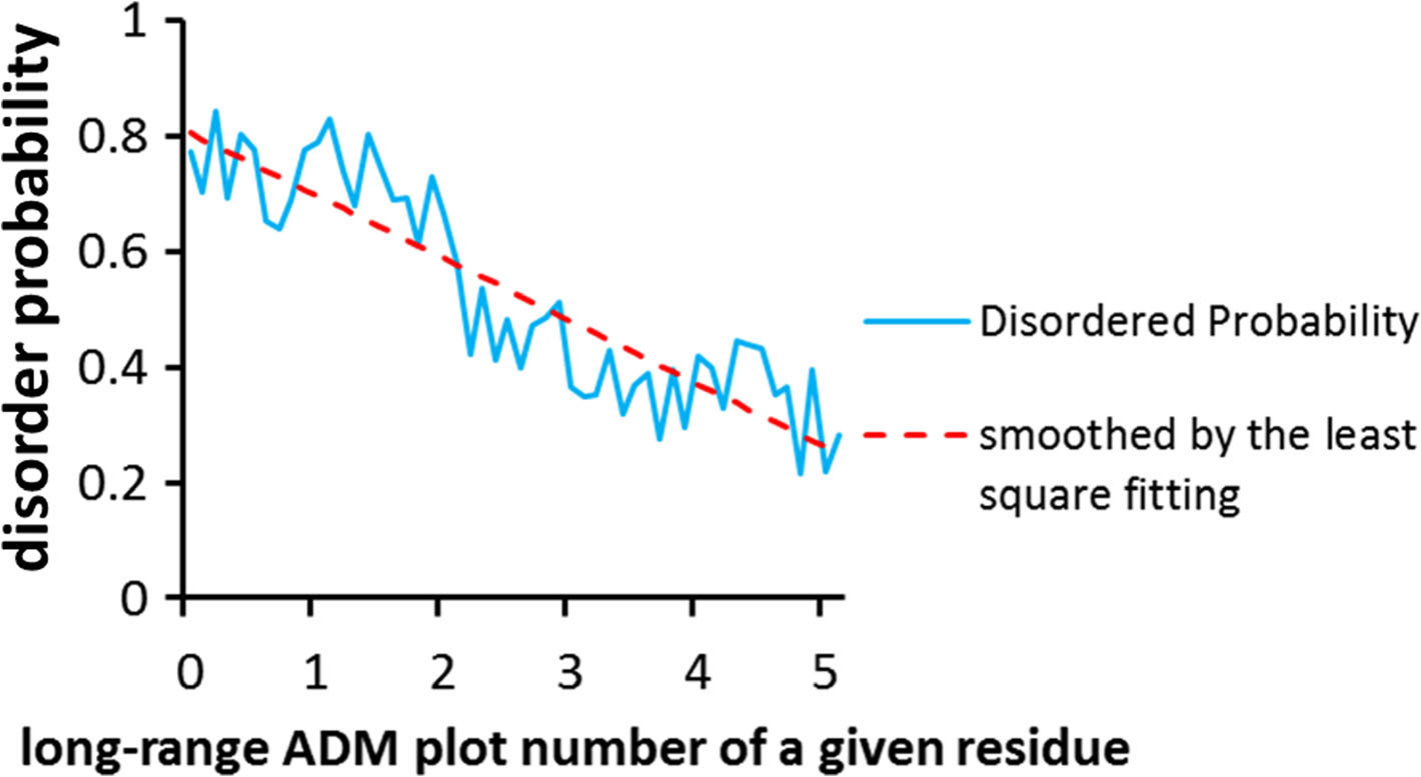
Plot of the ADM plot number of one residue (x-axis) vs. the probability that a residue is included in a disordered (ordered) region, that is, the disorder probability (y-axis). The profile is indicated by a blue line. The red broken line denotes the smoothed profile of the disorder probability plot by least squares fitting

In total, 50 partially disordered proteins with around 100 to 199 residues were selected from DisProt (Table 1 and Additional file 2: Table S2). Any protein in which the disordered regions cover less than 10% of the whole protein or the ordered regions cover less than 10% of the whole protein is excluded from the present study. The ADM plot number of each residue in the ADM for each protein was calculated, and the ADM plot numbers were smoothed by taking the average of 5 residues forward and backward from a selected residue. For 50 proteins, the statistics of the ADM plot number of a given residue, in particular, the statistics of whether a residue is included in an ordered or disordered region, was collected. Figure 5 shows these statistics, where the x- and y-axes indicate the ADM plot number of a residue and the probability that the residue is included in a disordered region as predicted from the statistics. We call this probability the “disorder probability”. This profile is smoothed by least squares fitting. The result is shown in Fig. 5 as a red broken line.

### Attempt to predict IDRs

In this section, we describe a technique for predicting IDRs in a given protein based on long-range ADM plots, using the values indicated by the red broken line in Fig. 5.

#### Determining the threshold of disorder probability to predict IDRs

We use the following accuracy criteria to judge whether a residue is included in an ordered region or disordered region based on the disorder probability.

$$ \mathrm{AC}{\mathrm{C}}_p=\frac{TP+ TN}{TP+ TN+ FP+ FN} $$

$$ \mathrm{AC}{\mathrm{C}}_w=\frac{1}{2}\left(\frac{TP}{TP+ FN}+\frac{TN}{TN+ FP}\right). $$

**Fig. 6:**
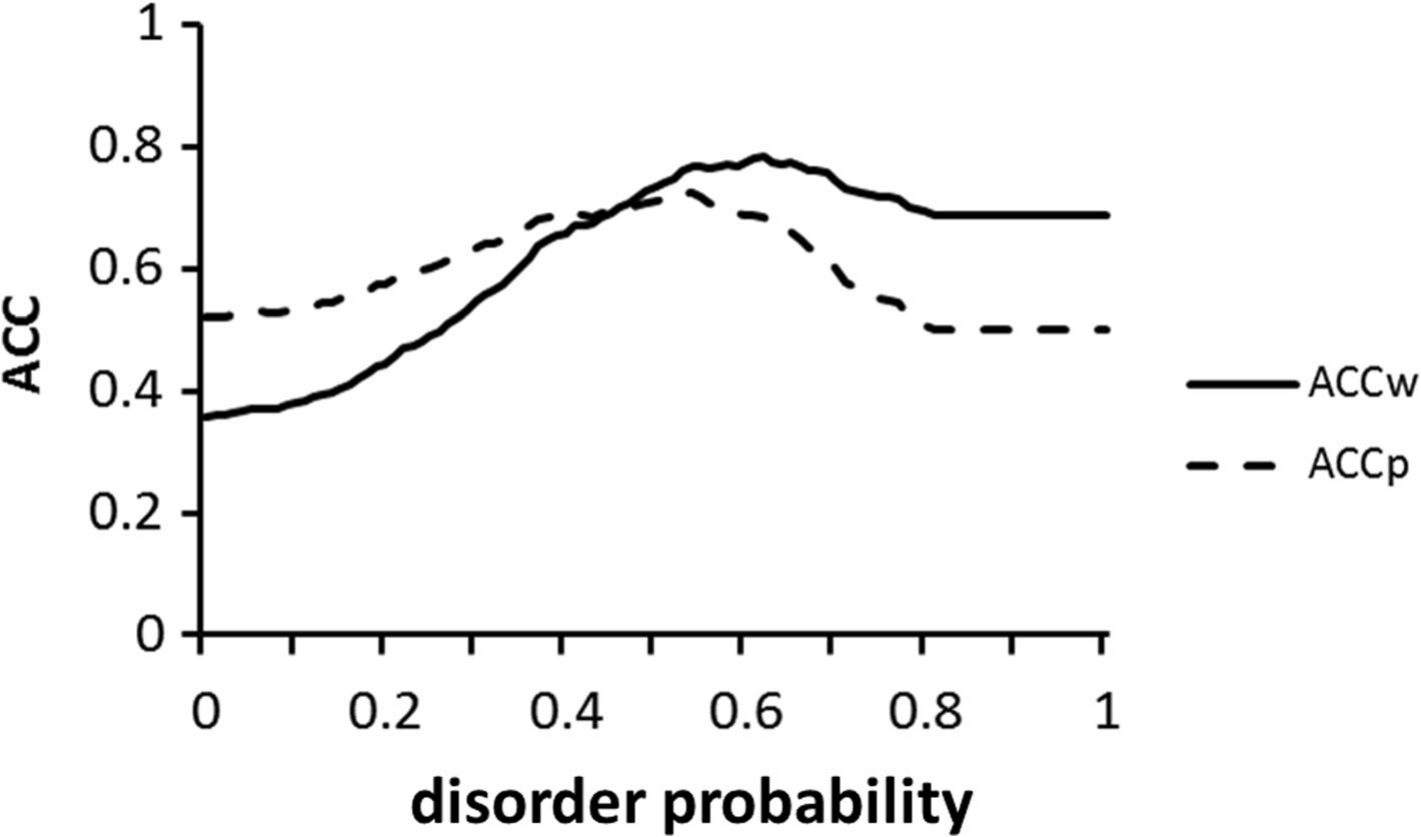
Relationship between disorder probability and ACC. A disorder probability of 0.53 shows the highest ACCp, 0.723, and a disorder probability of 0.62 provides the highest ACCw, 0.782

Here, TP refers to the number of residues in both predicted and actual disordered regions, that is, true positive. Similarly, FN, TN, and FP indicate the number of residues in predicted ordered regions that are actually in disordered regions, false negative (FN); the number of residues in predicted ordered and actual ordered regions, true negative (TN); and the number of residues in predicted disordered regions that are actually in ordered regions, false positive (FP). It should be noted that ACC_w_ indicates the accuracy of the prediction for partially disordered and partially ordered regions for the whole sequence, whereas ACCp emphasizes the accuracy for the prediction of partially disordered regions. Next, we attempted to predict the partially disordered regions for 10 newly selected partially disordered proteins from IDEAL, a database of IDPs, and compared the predictions and the actual locations of disordered regions. Then, ACC_w_ and ACCp were calculated (10 IDPs from *Homo sapiens* were selected from each category of Table1, with balanced contents of IDRs (Additional file 3: Table S3). The results are presented in Fig. 6. A value of 0.53 for the disorder probability shows the highest ACCp, 0.723, whereas a value of 0.62 for the disorder probability shows the highest ACCw, 0.782. Thus, we use 0.62 for the disorder probability as the threshold to improve the accuracy of the prediction of partially disordered regions.

#### Test of the present technique compared to other techniques

In this section, we examine whether our threshold works for 6 test disordered proteins from IDEAL (Table 2), using a data set consisting of 50 arbitrarily selected proteins from DisProt (Table 1 and Additional file 1: Table S1) with 100 to 199 residues. The 6 proteins presented in Table 2 were chosen so as not to include the previous 10 proteins used to determine the threshold with the same criteria.

**Table 2:** The 6 Proteins from IFDEAL used to test the present technique and for the comparison with the other 3 techniques

IID00378	small ubiquitin-related modifier 1 (*Homo sapiens*)
IID90012	histone H3K27 methylase (*Paramecium bursaria Chlorella virus* 1)
IID00019	histone H2A.Z (*Homo sapiens)*
IID00346	microtubule-associated proteins 1A/1B light chain 3B (*Homo sapiens*)
IID00272	histone H3-like centromeric protein A (*Homo sapiens*)
IID00186	baculoviral IAP repeat-containing protein 5 (*Homo sapiens*)

**Fig. 7:**
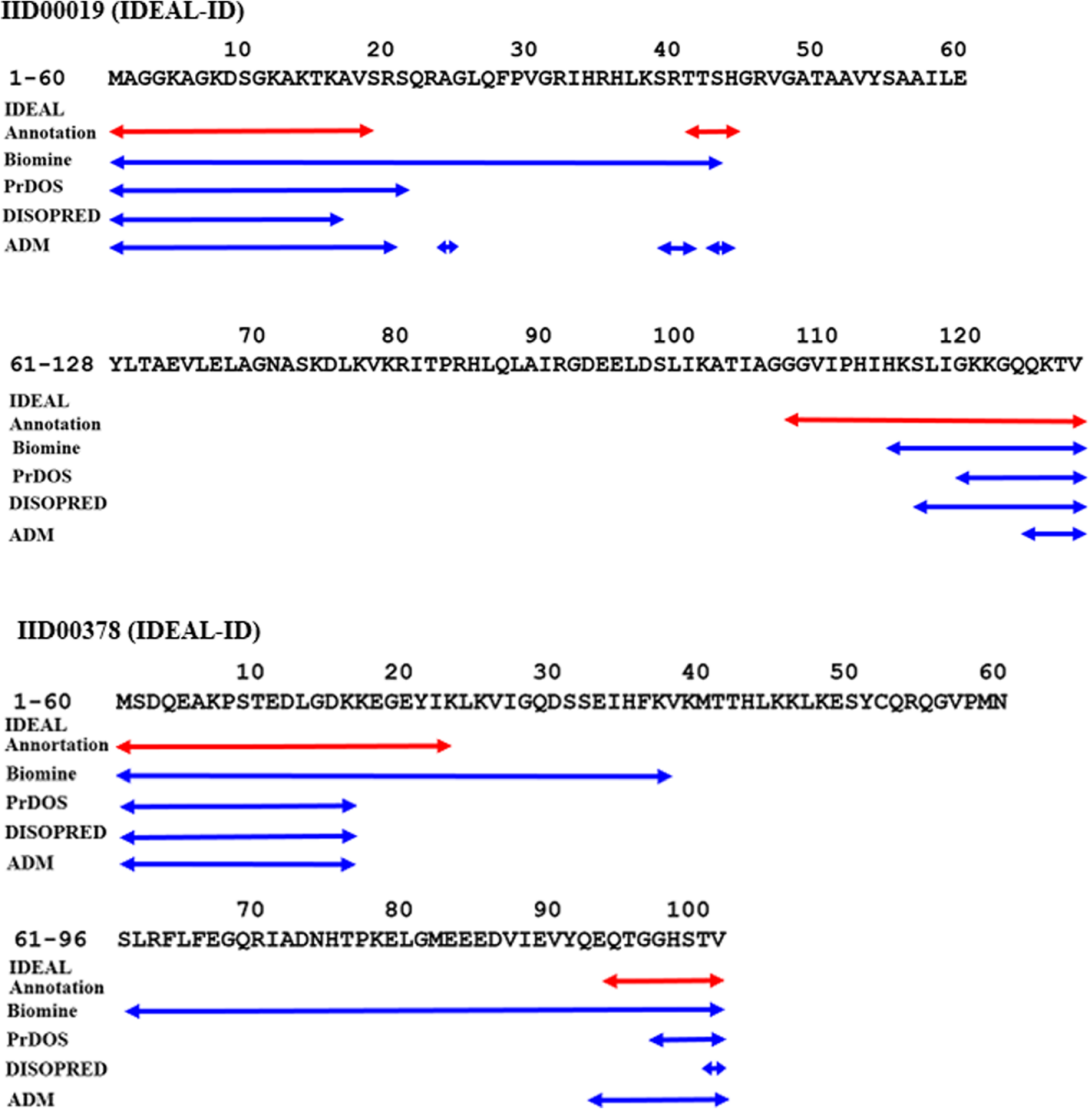
The sequences and comparisons of the results of prediction techniques (Biomine, PrDOS, DISOPRED, and the ADM technique in the present study) with the IDEAL annotations of Histone H2A.Z from *Homo sapiens* (IDEALID: IID00019) and small ubiquitin-related modifier 1 from *Homo sapiens* (IDEALID: IID00378). A segment with a red double arrow means the position of IDR annotated in IDEAL. A blue double arrow means a segment predicted as IDR by biomine, PrDOS, Disopred, or the ADM technique in the present study

The results for the predictions of the IDR positions are presented in Fig. 7 and (Additional file 4: Figure S1). The results obtained are compared with some techniques attempted in CASP10 competition. The techniques in CASP10 exhibit similar accuracy [12]. Among them, we chose the following three techniques, that is, Biomine [21], DISOPRED [22] and PrDOS [23]. These three techniques achieved the highest accuracies in the Matthews correlation coefficient (MCC) and are available on line. In Fig. 7, we show the results for just IID00019 and IID00378, that is, the sequences and comparisons of the results of prediction techniques (Biomine, PrDOS, DISOPRED, and the ADM technique in the present study) with the IDEAL annotations of Histone H2A.Z from *Homo sapiens* (IDEALID: IID00019) and small ubiquitin-related modifier 1 from *Homo sapiens* (IDEALID: IID00378). One interesting point is that for histone H2A.Z from *Homo sapiens* (IDEAL-ID: IID00019), the present technique predicts the position of the very short IDR at the part of the partial sequence “SRTTS”, corresponding to 4 residues, “TTSH” at 41–44, which was predicted as disordered by IDEAL. It is also interesting that PrDOS, DISOPRED, and the present ADM technique make the same prediction for the N-terminal IDR in small ubiquitin-related modifier 1 from *Homo sapiens* (IDEAL-ID: IID00378).

Table 3 presents the results for ACCw and ACCp obtained by the present ADM technique and the three other techniques. ACCw and ACCp are 0.793 using Biomine, 0.894 and 0.817 using PrDOS, and 0.902 and 0.795 using DISOPRED. The present technique with ADM yields a ACCw and ACCp of 0.845 and 0.741, respectively. That is, the present technique achieved a prediction accuracy that is comparable with the top three techniques in the CASP10 contest.

**Table 3:** Comparisons of the prediction accuracy of the present ADM technique and the top 3 techniques in the CASP10 experiment

	Biomine	PrDOS	DISOPRED	ADM
ACCw	0.79	0.89	0.90	0.85
ACCp	0.79	0.82	0.80	0.74

### Application to a sequence showing the IDP property but forming ordered structures when two sequences form a dimer

**Fig. 8:**
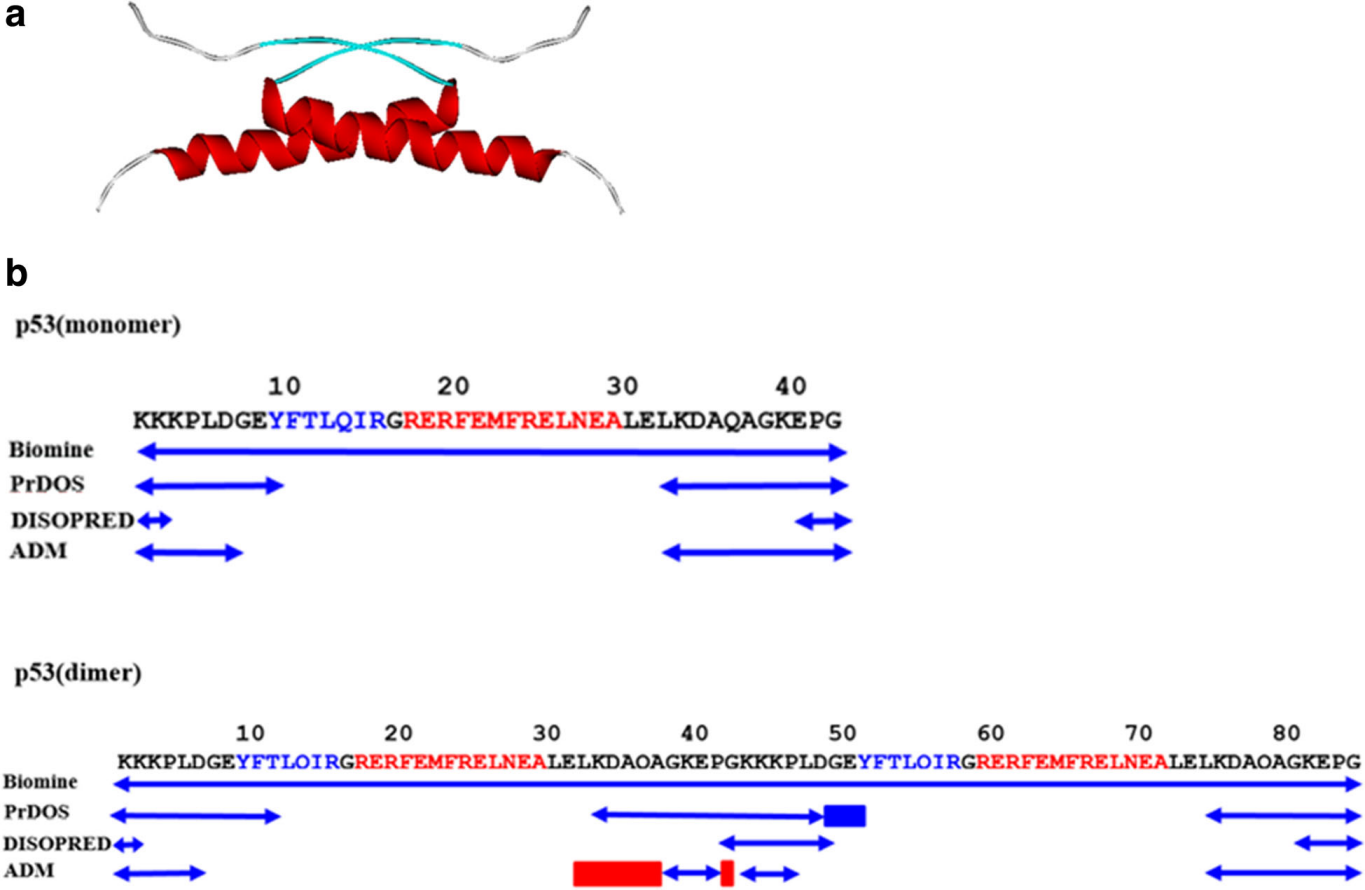
**a** Dimer part of p53 tetramerization domain. Monomer of p53 exhibits one α-helix and one β-strand. **b** The sequences and comparisons of the results of prediction techniques for the monomer and the dimer. The positions of an α-helix and a β-strand are indicated by red and blue letters in sequences p53 (monomer) and p53 (dimer). A segment with a red double arrow means the position of IDR. A blue double arrow means a segment predicted as IDR by Biomine, PrDOS, DISOPRED, or the ADM technique in the present study. It is assumed that the monomer exhibits the IDP property and the dimer exhibits ordered structures [24]. A red rectangle in the PrDOS and ADM predictions denotes the parts which show the predicted ordered regions only in the dimer

It is quite interesting to see the results when the present technique is applied to the protein p53, which shows the IDP property as a monomer, but its dimer formed by two p53 sequences exhibits ordered structures [24]. p53 protein is described in the structure with PDB ID: 3SAK. The dimer part of the p53 tetramerization domain is shown in Fig. 8(a). The monomer of p53 exhibits one α-helix strand and one β-strand.

The protein p53 is a tumor suppressor protein and the most significant hub protein in the network of cell cycle regulation proteins against genotoxic stress. That is, p53 is activated by genotoxic stress and works as a transcription factor to promote the transcription of downstream genes and induce cell cycle arrest and apoptosis. It is known that the tetramerization of p53 is indispensable for expression of the function [25].

Table 4 presents the results for p53 obtained by the present ADM technique and the three other techniques mentioned above.

**Table 4:** Comparisons of the prediction accuracy of the present ADM technique and the top 3 techniques in the CASP10 experiment for p53

	Miomine	PrDOS	DISOPRED	ADM
monomer	1.0	0.48	0.14	0.43
dimer	0.0	0.54	0.81	0.69

We apply the techniques for the p53 sequence itself and the sequence connecting two p53 sequences. The property of the sequence connecting two p53 sequences is not exactly same as that of the dimer. However, the results for the sequence connecting two p53 sequences are expected to show some properties of ordered structure formation. In the calculations of ACC, we assume that the p53 monomer exhibits complete disorder and that the dimer exhibits complete order. The same sequence of 3SAK in PDB is used. 3SAK is a tetramer of p53. According to DISOPRED, the major part of the sequence tends to be predicted as ordered for the monomer and dimer sequences, and thus the accuracy for the dimer tends to be high. The Biomine technique predicts the opposite, that the full sequences of both the monomer and dimer are disordered. Therefore, high ACC is observed for the monomer, but for the dimer, ACC is low. These results imply that DISOPRED and Biomine predict whether a given residue in a protein is in a disordered region or ordered region based on the properties of the local residues around the amino acid considered.

In contrast, the present ADM technique and PrDOS made predictions with high accuracy for both the monomer and dimer, as shown in Table 4. The present technique achieves slightly better accuracy. Figure 8(b) presents the predicted ordered regions or disordered regions of the p53 monomer and dimer sequences and compares the predicted regions with the actual regions (see above). Our ADM technique shows an extension of the ordered region in the C-terminus of the first p53 sequence in the dimer, namely, 32–37 and 42 [enclosed by the red dotted rectangles in Fig. 8(b)], while a slight extension in the N-terminus of the second p53 sequence is observed, that is, 49–51 in the result by PrDOS, as shown in Fig. 8(b) (blue dotted rectangle). Research suggests that the helix part of the p53 monomer tends to form ordered structure during molecular dynamics simulations [26]. In a system such as p53, a sufficient hydrophobic core is not formed within a monomer, and the p53 monomer behaves as an IDP; but by forming a dimer, a definite hydrophobic core is formed, and the p53 dimer exhibits ordered structures [27].

The results of the present technique reflect this, because ADM includes the effect of long-range inter-residue interactions along a sequence. The same effect is considered to be incorporated in PrDOS. Therefore, the present ADM technique may be able to predict the conformational change from disorder to order induced by the polymerization of IDP sequences and vice versa.

## Discussion

In the present study, we introduced a new technique for predicting IDRs by means of maps based on inter-residue average distance statistics, average distance maps (ADM). The accuracy of the present technique is comparable to the techniques in the CASP10 contest with relatively high achievement including PrDOS, DISOPRED, and Biomine.

In the prediction for the p53 dimer, the accuracy of the present technique is the highest compared to current ways of making predictions. Our ADM technique is expected not only to predict IDRs but also the formation of ordered structures by dimerization.

Our ADM technique may predict a core region of IDPs forming definite 3D structures through interactions, such as dimerization and so on. Furthermore, our study may serve to predict a disordered region which would become an ordered structure when binding to another protein.

## Conclusion

The present study demonstrates that a new technique based on the average distance map (ADM) can provide a prediction of intrinsically disordered regions in a protein in good accuracy. This method can be applied to a protein which shows a disorder property as a monomer but ordered character when its form a dimer. These results suggest that the inter-residue average distance statistics includes various properties of proteins.

## Additional files

Additional file 1: **Table S1.** List of the proteins used as the data set (DOCX 22 kb)

Additional file 2: **Table S2.** Partially disordered proteins from DisProt used for the determination of disorder probability (DOCX 18 kb)

Additional file 3: **Table S3.** Proteins used for the determination of the ACC threshold value (DOCX 15 kb)

Additional file 4 **Figure S1.** Comparisons of prediction technique results (Biomine, PrDOS, DISOPRED, and the ADM technique from the present study) in terms of amino acid sequences for The baculoviral IAP repeat-containing protein 5 from *Homo sapiens* (IDEALID: IID00186), histone H3-like centromeric protein A from *Homo sapiens* (IDEALID: IID00272), microtubule-associated proteins 1A/1B light chain 3B from *Homo sapiens* (IDEALID: IID00346), and histone H3K27 methylase from *Paramecium bursaria Chlorella virus 1* (IDEALID: IID90012). A segment with white letters on the black background refers to the position of IDR as annotated in DisProt and predicted by a technique used in this study. (DOCX 170 kb)

## Abbreviations

ADM: Average distance map
CASP: Critical Assessment of protein Structure Prediction
CBP: CREB-binding protein
CREB: Cyclic-AMP response element-binding protein
IDP: Intrinsically disordered protein
IDR: Intrinsically disordered region
PDB: Protein Data Bank
