# Supplementary material for: A new technique for predicting intrinsically disordered regions based on average distance map constructed with inter-residue average distance statistics
Source: BMC Struct Biol. 2019 Feb 6;19:3. doi: 10.1186/s12900-019-0101-3 (PMC6366092; doi:10.1186/s12900-019-0101-3)
Supplement: Supplementary file 1 — Table S1. List of the proteins used as the data set (DOCX 22 kb) [file 12900_2019_101_MOESM1_ESM.docx]

Additional File 1

Table S1. List of proteins used as the dataset

|  | 100% disordered proteins from DisProt | | | partially disordered proteins from DisProt | | | 100% ordered proteins from PDB | | |
| --- | --- | --- | --- | --- | --- | --- | --- | --- | --- |
| 60-79 | DP00146 | DP00617 |  | DP00015 | DP00416 |  | 1COK | 1MHX | 1WCN |
|  | DP00158 |  |  | DP00042 | DP00529 |  | 1FVS | 1RUW | 1Z7T |
|  | DP00180_C003 |  |  | DP00130 | DP00543 |  | 1G2B | 1T1T | 2L2D |
|  | DP00216 |  |  | DP00137 |  |  | 1G2S | 1T7A | 2LT1 |
|  | DP00288 |  |  | DP00179 |  |  | 1G25 | 1V66 | 2MO0 |
|  | DP00586 |  |  | DP00202 |  |  | 1HYI | 1V80 | 2RTS |
|  | DP00592 |  |  | DP00277 |  |  | 1ITP | 1W4I |  |
| 80-99 | DP00022 | DP00347 |  | DP00296 | DP00511 |  | 1DVV | 1TZ1 | 2GV1 |
|  | DP00024 | DP00359 |  | DP00373 | DP00573_C003 |  | 1EMN | 1U38 | 2JOY |
|  | DP00027 | DP00465 |  | DP00388 | DP00638 |  | 1FBR | 1V5R | 2JYG |
|  | DP00039 | DP00510 |  | DP00412 | DP00641 |  | 1JRU | 1WZ6 | 2MW5 |
|  | DP00140 | DP00665 |  | DP00422 | DP00685 |  | 1RHW | 1X4E | 2MX2 |
|  | DP00147 |  |  | DP00432 | DP00696 |  | 1SG5 | 1X4Q | 3P63 |
|  | DP00242 |  |  | DP00509 | DP00701 |  | 1TXE | 2CWY |  |
| 100-119 | DP00001 | DP00058 | DP00531 | DP00113 | DP00382 | DP00661 | 1CRG | 1QBH | 1TTO |
|  | DP00002 | DP00069 | DP00532 | DP00125 | DP00424 | DP00673_C001 | 1CWW | 1R57 | 1TTV |
|  | DP00005 | DP00164 | DP00538 | DP00201 | DP00513 |  | 1E2B | 1SM7 | 1U2N |
|  | DP00006 | DP00185 | DP00550 | DP00289 | DP00527 |  | 1IIY | 1T00 | 1WX7 |
|  | DP00028 | DP00205 | DP00626_C001 | DP00292 | DP00567 |  | 1JI8 | 1T2J | 2KQ7 |
|  | DP00038 | DP00372 | DP00650 | DP00299 | DP00622 |  | 1JWE | 1TE7 | 5FRG |
|  | DP00040 | DP00387 |  | DP00380 | DP00637 |  | 1P1L | 1TFT |  |
| 120-139 | DP00145 |  |  | DP00009 | DP00643_A002 |  | 1A5P | 1RZW | 2NBO |
|  | DP00219 |  |  | DP00263 | DP00709 |  | 1ACF | 1S3D | 2QHE |
|  | DP00534 |  |  | DP00276 |  |  | 1DT1 | 1T8V | 2UX6 |
|  | DP00544 |  |  | DP00340 |  |  | 1E8L | 1U89 | 2X6M |
|  | DP00555 |  |  | DP00501 |  |  | 1HPW | 1V49 | 3DIH |
|  | DP00626 |  |  | DP00512 |  |  | 1I3U | 1VC8 | 4PS6 |
|  | DP00630 |  |  | DP00551 |  |  | 1QD0 | 1ZHV |  |
| 140-159 | DP00070 |  |  | DP00013 | DP00344 | DP00518 | 1DF7 | 1V9Q | 2LT2 |
|  | DP00112 |  |  | DP00067 | DP00350 | DP00570 | 1GOC | 1WFR | 2ML1 |
|  | DP00174 |  |  | DP00084_A002 | DP00393 | DP00652 | 1GWM | 1WJ1 | 2RRK |
|  | DP00186 |  |  | DP00206 | DP00414 | DP00674_C001 | 1H6H | "2E34" | 2V1A |
|  | DP00303 |  |  | DP00259 | DP00415 | DP00676 | 1RCY | 2HD9 | 3JZZ |
|  |  |  |  | DP00301 | DP00457 | DP00677 | 1SRA | 2K7H | 5I12 |
|  |  |  |  | DP00337 | DP00504 |  | 1UWF | 2LPE |  |
| 160-179 | DP00047 | DP00564 |  | DP00084 | DP00335 | DP00482 | 1AX3 | 1TP9 | 2LRP |
|  | DP00193 | DP00663 |  | DP00106 | DP00336 | DP00505 | 1BXD | 1TVJ | 2LUO |
|  | DP00200 | DP00664 |  | DP00194 | DP00358 | DP00588_C002 | 1CLH | 1TYJ | 4AXQ |
|  | DP00237 |  |  | DP00249 | DP00434 | DP00619 | 1D5C | 1WS6 | 4E2U |
|  | DP00325 |  |  | DP00254 | DP00444 | DP00624 | 1DFF | 1YYC | 4O6G |
|  | DP00530 |  |  | DP00255 | DP00445 | DP00693 | 1MJN | 2HO9 | 4Y0A |
|  | DP00546 |  |  | DP00279 | DP00469 | DP00704 | 1RKB | 2LQ8 |  |
| 180-199 | DP00560_C007 |  |  | DP00018 | DP00197 | DP00508 | 1A23 | 1QCY | 1VDX |
|  | DP00658 |  |  | DP00044 | DP00257 | DP00583 | 1EH1 | 1QF9 | 1Y1A |
|  | DP00667 |  |  | DP00097 | DP00290 | DP00645 | 1EQ6 | 1RW5 | 2H5C |
|  | DP00689 |  |  | DP00103 | DP00306 | DP00668 | 1FY9 | 1T0V | 2YWO |
|  |  |  |  | DP00153 | DP00364 | DP00669 | 1IJ9 | 1T68 | 4ARH |
|  |  |  |  | DP00167 | DP00408 |  | 1JIG | 1U3B | 4DVC |
|  |  |  |  | DP00192 | DP00438 |  | 1P1D | 1U17 |  |
| 200-219 | DP00041 | DP00563 |  | DP00029 | DP00247 | DP00575 | 1AEC | 1K7J | 1Z77 |
|  | DP00068 |  |  | DP00048 | DP00287 | DP00581 | 1AUN | 1NHY | 2E1B |
|  | DP00232 |  |  | DP00063 | DP00302 | DP00600 | 1AUQ | 1OMR | 2IUE |
|  | DP00330 |  |  | DP00142 | DP00384 | DP00607 | 1BF8 | 1Q1P | 2KS6 |
|  | DP00367 |  |  | DP00166 | SP00506 | DP00687 | 1CFB | 1U0A | 3KBB |
|  | DP00421 |  |  | DP00189 | SP00552 | DP00710 | 1ENF | 1U9Q | 3ZPM |
|  | DP00521 |  |  | DP00208 | DP00573_C006 |  | 1IJB | 1WD5 |  |
