# Supplementary material for: A new technique for predicting intrinsically disordered regions based on average distance map constructed with inter-residue average distance statistics
Source: BMC Struct Biol. 2019 Feb 6;19:3. doi: 10.1186/s12900-019-0101-3 (PMC6366092; doi:10.1186/s12900-019-0101-3)
Supplement: Supplementary file 2 — Table S2. Partially disordered proteins from DisProt used for the determination of disorder probability (DOCX 18 kb) [file 12900_2019_101_MOESM2_ESM.docx]

Additional File 2

Table S2. Partially disordered proteins from DisProt used for the determination of disorder probability

| DP00113 | DP00661 | DP00393 | DP00194 | DP00624 |
| --- | --- | --- | --- | --- |
| DP00125 | DP00673 | DP00414 | DP00249 | DP00704 |
| DP00201 | DP00276 | DP00415 | DP00255 | DP00044 |
| DP00289 | DP00512 | DP00457 | DP00279 | DP00257 |
| DP00380 | DP00551 | DP00504 | DP00335 | DP00290 |
| DP00382 | DP00643_A002 | DP00518 | DP00358 | DP00306 |
| DP00424 | DP00709 | DP00652 | DP00469 | DP00408 |
| DP00527 | DP00013 | DP00676 | DP00482 | DP00438 |
| DP00622 | DP00067 | DP00084 | DP00505 | DP00508 |
| DP00637 | DP00084_A002 | DP00106 | DP00588 | DP00669 |
