# Supplementary material for: A new technique for predicting intrinsically disordered regions based on average distance map constructed with inter-residue average distance statistics
Source: BMC Struct Biol. 2019 Feb 6;19:3. doi: 10.1186/s12900-019-0101-3 (PMC6366092; doi:10.1186/s12900-019-0101-3)
Supplement: Supplementary file 3 — Table S3. Proteins used for the determination of the ACC threshold value (DOCX 15 kb) [file 12900_2019_101_MOESM3_ESM.docx]

Additional File 3

Table S3. Proteins used for determination of threshold value of ACC

| IID00010 | IID00058 | IID00060 | IID00308 | IID00206 |
| --- | --- | --- | --- | --- |
| IID00056 | IID00059 | IID00274 | IID00161 | IID00238 |
