# Supplementary material for: A new technique for predicting intrinsically disordered regions based on average distance map constructed with inter-residue average distance statistics
Source: BMC Struct Biol. 2019 Feb 6;19:3. doi: 10.1186/s12900-019-0101-3 (PMC6366092; doi:10.1186/s12900-019-0101-3)
Supplement: Supplementary file 4 — Figure S1. Comparisons of prediction technique results (Biomine, PrDOS, DISOPRED, and the ADM technique from the present study) in terms of amino acid sequences for The baculoviral IAP repeat-containing protein 5 from Homo sapiens (IDEALID: IID00186), histone H3-like centromeric protein A from Homo sapiens (IDEALID: IID00272), microtubule-associated proteins 1A/1B light chain 3B from Homo sapiens (IDEALID: IID00346), and histone H3K27 methylase from Paramecium bursaria Chlorella virus 1 (IDEALID: IID90012). A segment with white letters on the black background refers to the position of IDR as annotated in DisProt and predicted by a technique used in this study. (DOCX 170 kb) [file 12900_2019_101_MOESM4_ESM.docx]

Additional File 4

Figure S1: The sequences and comparisons of the results of prediction techniques (biomine, PrDOS, Disopred, and the present study) with IDEAL annotations for Baculoviral IAP repeat-containing protein 5 from *Homo sapiens* (IDEAL-ID: IID00186), Histone H3-like centromeric protein A from *Homo sapiens* (IDEAL-ID: IID00272), Microtuble-associated proteins 1A/1B light chain 3B from *Homo sapiens* (IDEAL-ID: IID00346) and Histone H3K27 methylase from *Paramecium bursaria Chlorella virus 1* (IDEAL-ID: IID90012). A segment with a red double arrow means the position of IDR annotated in IDEAL and blue double arrow means a segment predicted as IDR by biomine, PrDOS, Disopred, or the present study.


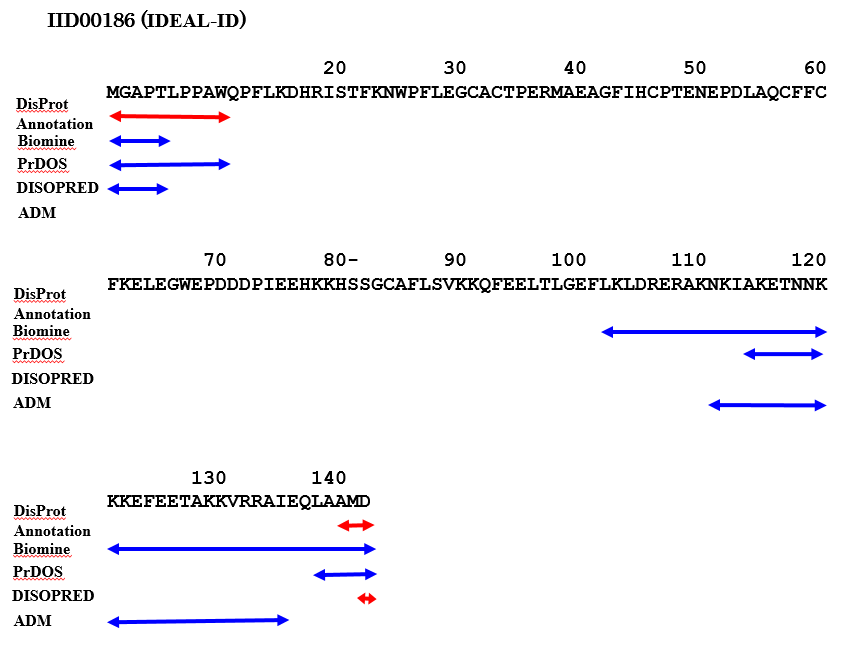


**IDEAL**

**Annotation**

**IDEAL**

**Annotation**

**IDEAL**

**Annotation**

(continued)


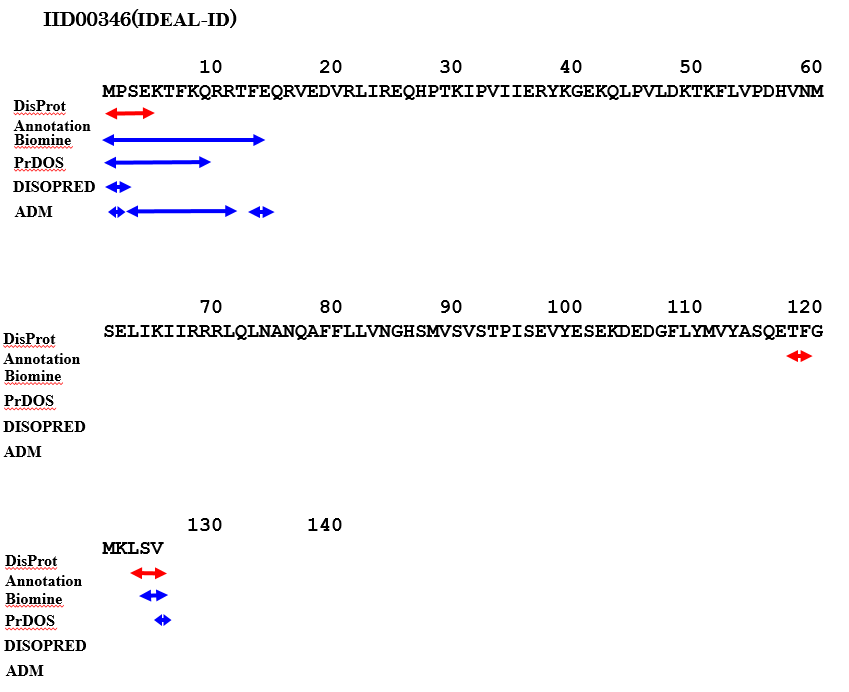


**IDEAL**

**Annotation**

**IDEAL**

**Annotation**

**IDEAL**

**Annotation**

(continued)


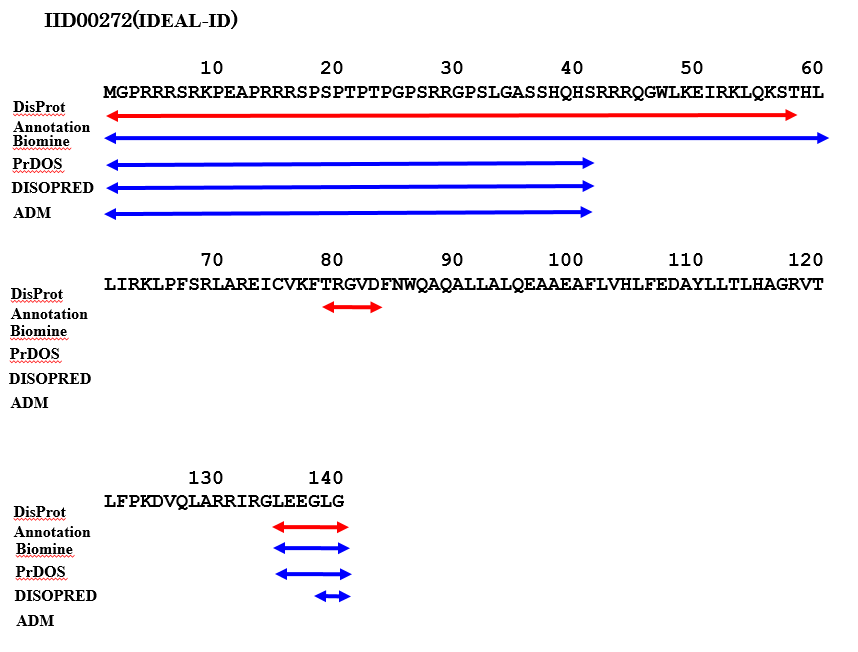


**IDEAL**

**Annotation**

**IDEAL**

**Annotation**

**IDEAL**

**Annotation**

(continued)


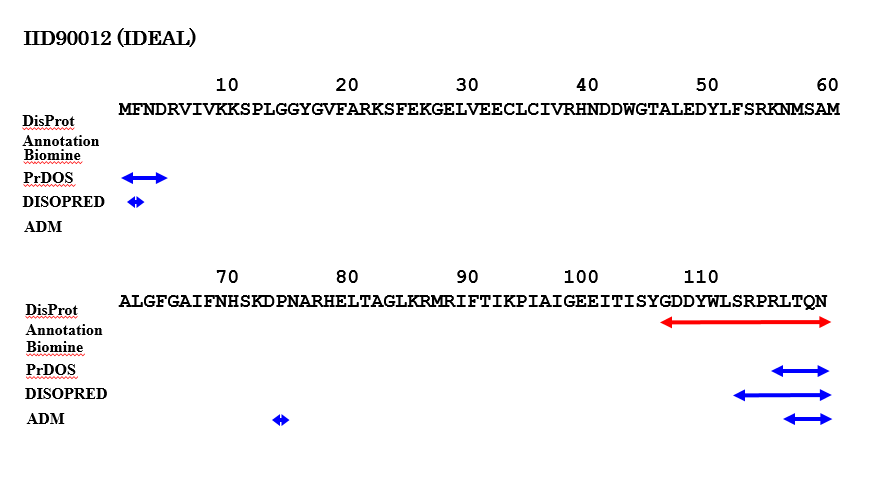


**IID90012 (IDEAL-ID)**

**IDEAL**

**Annotation**

**IDEAL**

**Annotation**
